# A Multi-Serotype Approach Clarifies the Catabolite Control Protein A Regulon in the Major Human Pathogen Group A *Streptococcus*

**DOI:** 10.1038/srep32442

**Published:** 2016-09-01

**Authors:** Sruti DebRoy, Miguel Saldaña, Dante Travisany, Andrew Montano, Jessica Galloway-Peña, Nicola Horstmann, Hui Yao, Mauricio González, Alejandro Maass, Mauricio Latorre, Samuel A. Shelburne

**Affiliations:** 1 Department of Infectious Diseases, MD Anderson Cancer Center, Houston, Texas, USA; 2 Mathomics, Center for Mathematical Modeling, Universidad de Chile, Beauchef 851, 7th Floor, Santiago, Chile; 3 Center for Genome Regulation (Fondap 15090007), Universidad de Chile, Blanco Encalada 2085, Santiago, Chile; 4 Department of Bioinformatics and Computational Biology, MD Anderson Cancer Center, Houston, Texas, USA; 5 Laboratorio de Bioinformática y Expresión Génica, INTA, Universidad de Chile, El Líbano 5524, Macul, Santiago, Chile; 6 Department of Mathematical Engineering, Universidad de Chile, Beauchef 851, 5th Floor, Santiago, Chile; 7 Department of Genomic Medicine, MD Anderson Cancer Center, Houston, Texas, USA

## Abstract

Catabolite control protein A (CcpA) is a highly conserved, master regulator of carbon source utilization in gram-positive bacteria, but the CcpA regulon remains ill-defined. In this study we aimed to clarify the CcpA regulon by determining the impact of CcpA-inactivation on the virulence and transcriptome of three distinct serotypes of the major human pathogen Group A *Streptococcus* (GAS). CcpA-inactivation significantly decreased GAS virulence in a broad array of animal challenge models consistent with the idea that CcpA is critical to gram-positive bacterial pathogenesis. Via comparative transcriptomics, we established that the GAS CcpA core regulon is enriched for highly conserved CcpA binding motifs (i.e. *cre* sites). Conversely, strain-specific differences in the CcpA transcriptome seems to consist primarily of affected secondary networks. Refinement of *cre* site composition via analysis of the core regulon facilitated development of a modified *cre* consensus that shows promise for improved prediction of CcpA targets in other medically relevant gram-positive pathogens.

It is being increasingly appreciated that efficient acquisition and metabolism of nutrients are key aspects of bacterial pathogenesis^1^,^2^,^3^. Adaptation to various nutritional niches depends critically on the ability of bacteria to respond to diverse environmental stimuli^4^,^5^. Perception of these stimuli is relayed by regulatory proteins to effect changes at transcriptional and translational levels leading to adaptive changes at the cellular level^6^. Not surprisingly, therefore, regulatory proteins that affect basic bacterial physiology and metabolism have been found to be intimately linked to virulence in several infectious bacteria^7^,^8^,^9^. Carbon catabolite repression (CCR) is one of the most fundamental and highly conserved mechanisms which ensures optimal utilization of energy resources^10^. In many gram-positive bacteria, CCR is primarily mediated by the highly conserved catabolite control protein A (CcpA)^11^ which interacts with pseudo-palindromic *cis*-acting DNA motifs known as catabolite responsive elements (*cre*) to alter target gene expression^12^. In the presence of its phosphorylated co-effector histidine-containing protein HPr (HPr-Ser46-P), CcpA binds target DNA with increased affinity^13^. The production of HPr-Ser46-P by HPr kinase/phosphorylase (HPrK/P), in turn, is determined by the intra-cellular energy status, and thus the CcpA-(HPr-Ser46-P) interaction links CcpA-mediated transcription to changing nutritional conditions^14^.

Several studies have demonstrated a connection between CcpA and the virulence of major gram-positive pathogens such as *Streptococcus pyogenes*, *Streptococcus pneumoniae*, *Streptococcus mutans*, *Staphylococccus aureus*, *Clostridium difficile*, *Bacillus anthracis*, and *Enterococcus faecium*^15^,^16^,^17^,^18^,^19^,^20^,^21^,^22^,^23^,^24^,^25^. In light of the tremendous burden of gram-positive bacterial infections, CcpA is one of the most influential regulatory proteins modulating the virulence landscape of pathogenic bacteria^26^. Despite being extensively investigated for over 20 years, there remains numerous, critical knowledge gaps regarding CcpA function and its impact on bacterial pathogenesis. For example, although CcpA is known to affect expression of ~15% of the genomes of numerous bacterial species, the contribution of direct vs. indirect CcpA-mediated effects on transcription remains unclear^15^,^27^. Similarly, the role of CcpA in gram-positive bacterial virulence has primarily been studied in bacteremia models, which only represent a fraction of the types of infections that CcpA-containing bacteria cause^28^. Further information regarding the contribution of CcpA to gram-positive bacterial pathogenesis is needed given the potential role of the CcpA-(HPr-Ser46-P)-HPrK/P axis as a novel antimicrobial target^29^.

The majority of knowledge regarding CcpA-mediated effects on gene expression has occurred in the avirulent *Bacillus subtilis* which is distantly related to most organisms in which a role for CcpA in pathogenesis has been established^16^,^21^,^30^,^31^. In contrast, Group A *Streptococcus* (GAS) causes a wide range of infections in humans for which animal models are established^32^, is rendered less virulent by CcpA-inactivation, and is closely related to other organisms whose infectivity is affected by CcpA-deletion such as *S. pneumoniae* and *Streptococcus suis*^15^,^18^. Thus, GAS can be used to assess the nature of the regulatory impact of CcpA on the infectivity of gram-positive pathogens. We previously established that CcpA-inactivation in a serotype M1 GAS strain affects the expression of ~15% of the genome and reduces lethality in a bacteremia infection model. There are >140 distinct M serotypes of GAS and inactivation of a regulatory protein can have dramatically different effects depending on the GAS serotype being studied^33^,^34^. Thus, we sought to study CcpA-inactivation in multiple GAS serotypes to test the hypotheses that the effect of CcpA-inactivation on GAS virulence is not serotype dependent and that comparative transcriptomics of GAS strains lacking CcpA would facilitate identification of the core GAS CcpA regulon.

## Results and Discussion

### *CcpA* deletion has serotype-specific effects

**Figure 1:**
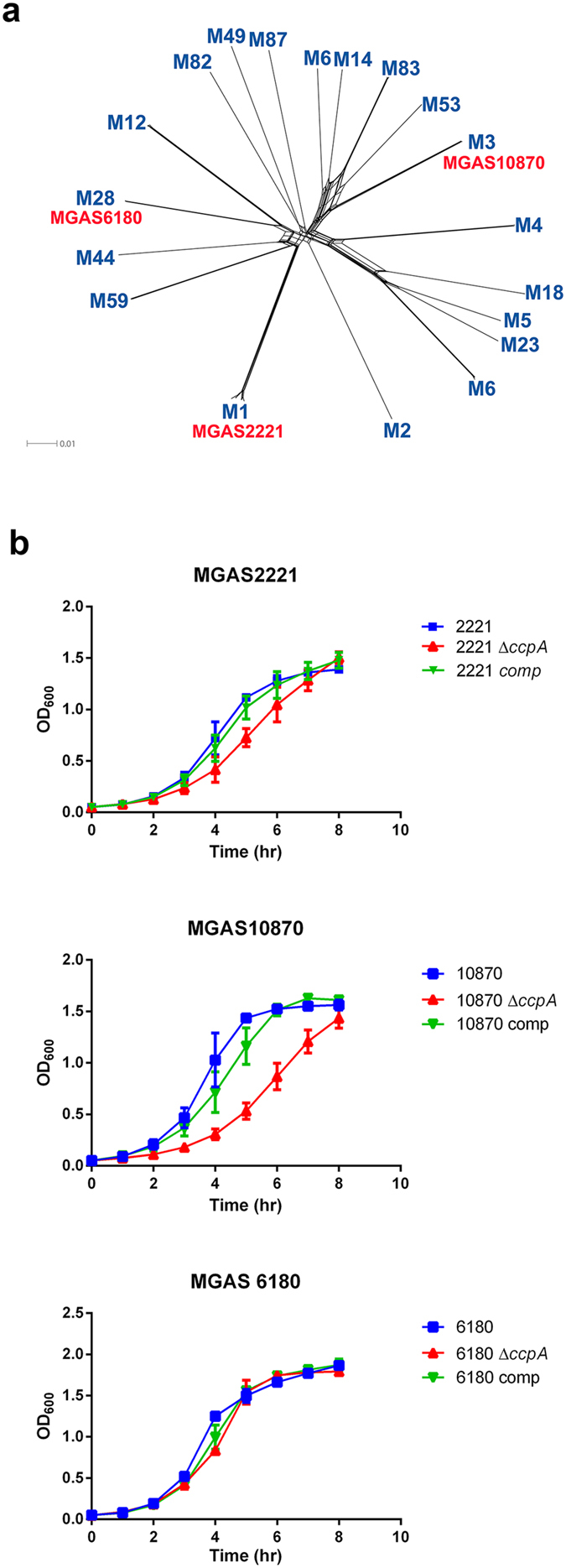
Characterization of the GAS M serotype strains and their *ccpA* derivatives. (**a**) Whole genome phylogeny of sequenced GAS strains. Complete GAS genomes of indicated M serotypes (blue) were obtained from NCBI. Relationships were inferred from 68,084 core single nucleotide polymorphism (SNP) loci using SplitsTree. Location of parental strains used in this work are shown in red. (**b**) Growth curves for wild type, *ccpA*-inactivated and complemented strains in THY. Data points are mean and standard deviations from duplicate samples of each strain measured on two independent occasions.

To improve understanding of the contribution of CcpA to the broad pathophysiology of GAS infections, we sought to analyze CcpA function in M serotype strains that are leading causes of GAS infections and are distantly related based on whole-genome phylogeny (Fig. 1a)^35^,^36^,^37^,^38^. We also used parental strains that are fully sequenced and known to lack mutations in the control of virulence (CovRS) two component system which affects the CcpA transcriptome because CovR and CcpA co-regulate numerous GAS genes^39^. Thus, we chose strains MGAS2221 (M1), MGAS10870 (M3), and MGAS6180 (M28) for our study^36^,^37^,^38^. The CcpA protein sequence in these three strains is identical (data not shown). Inactivation of CcpA in these three backgrounds resulted in a substantial growth defect in rich medium only for the serotype M3 strain MGAS10870 (Fig. 1b). Although why only the MGAS10870Δ*ccpA* strain shows a growth defect is not known, importantly, this growth defect is recovered in the *ccpA*-complemented strain (Fig. 1b) attributing the absence of *ccpA* as the primary cause of the growth defect. Consistent with previous observations for serotype M1 strains, CcpA-inactivation reduced colony size in strain MGAS10870^16^,^39^. Conversely, MGAS6180 colonies were uniformly small and unaffected by the loss of *ccpA* (Supplementary Fig. S1).

### CcpA affects GAS virulence in multiple infection models

**Figure 2:**
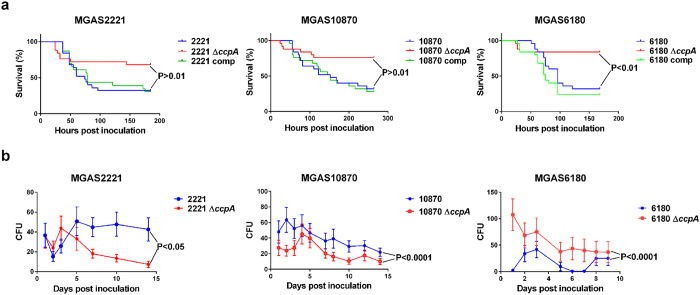
Effect of *ccpA* deletion on virulence of GAS serotype strains in mouse models of infection. CD-1 swiss mice were inoculated with GAS strains by intraperitoneal (**a**) or intranasal (**b**) route. Mice were monitored to near-mortality for the bacteremia model (**a**) and survival was graphed. For the oropharyngeal model (**b**), throat swabs were obtained daily and the bacterial density was graphed. P values were derived from repeated measures analysis (see Methods).

The major disease manifestations of GAS in humans are bacteremia, necrotizing fasciitis, skin/soft tissue infection, and pharyngitis, each of which have corresponding mouse models of infection^40^. Thus, we next assessed the contribution of CcpA to GAS virulence in each murine challenge model. Compared to their parental strains, all *ccpA*-inactivated strains caused significantly less mortality in the bacteremia model (Fig. 2a). We confirmed the lack of spurious mutations causing the observed differences by showing that complementation of the mutant strains with *ccpA in trans* restored virulence to wild-type levels (Fig. 2a). Similarly, in the myositis model, which mimics necrotizing fasciitis in humans, *ccpA*-inactivation significantly decreased mortality for all serotypes (Supplementary Fig. S2). Moreover, in the subcutaneous challenge model of skin/soft tissue infection, all three parental strains formed significantly larger ulcers than their *ccpA*-inactivated derivatives (Supplementary Fig. S2). Finally, following oropharyngeal challenge, a model for human pharyngitis, *ccpA*-inactivation caused a significant decrease in organism recovery for the M1 and M3 strains. However, we observed a significant increase in organism density over time for strain 6180Δ*ccpA* compared to MGAS6180 (Fig. 2b). One possible explanation for these discordant results is the known inability of MGAS6180 to produce the broad-spectrum cysteine protease SpeB, which inactivates innate effector molecules present in human saliva^41^. Indeed, we found that CcpA-inactivation significantly decreased SpeB activity in MGAS2221 and MGAS10870, but did not affect the absence of SpeB production in MGAS6180 (Supplementary Fig. S3). Thus, in 11 of 12 animal challenge studies, CcpA-inactivation significantly decreased GAS virulence consistent with the idea that CcpA is critical to GAS pathogenesis across a diverse array of serotypes and infection models.

### Using a multi-serotype approach to define the core GAS CcpA regulon

**Figure 3:**
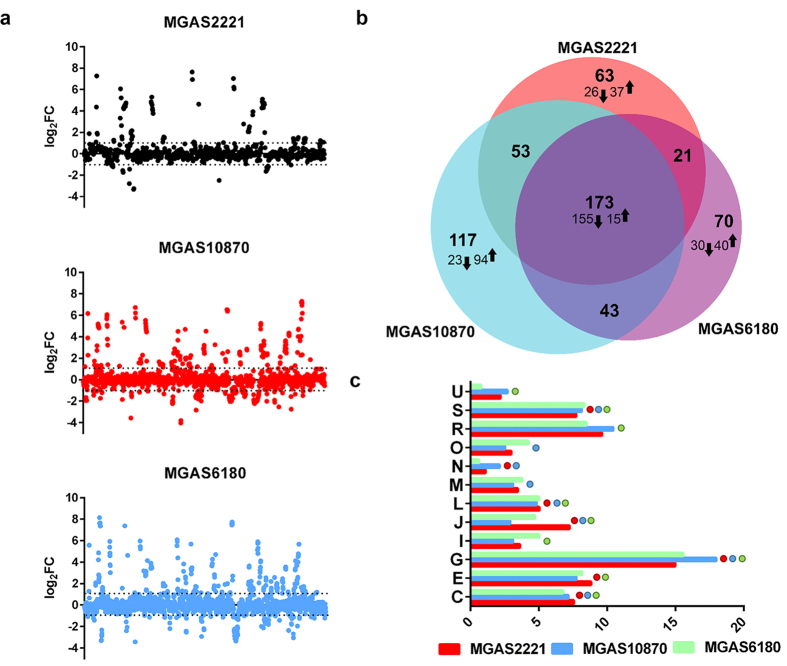
The effect of *ccpA* deletion on the GAS transcriptome. (**a**) Linear representation of the genome of GAS serotype strains showing the effect of *ccpA*-inactivation on gene expression depicted as log fold change. (**b**) A weighted Venn diagram displaying the distribution and overlap of the CcpA-affected genes in all three serotypes was generated using the BioVenn application^60^. Arrows indicate the number of genes repressed (down) and activated (up) by CcpA in individual serotypes and in the core regulon. (**c**) Genes affected by CcpA in each serotype are shown by order of COG categories. Each colored circle denotes the enrichment of a specific COG in the serotype strain. [C] Energy production and conversion; [E] Amino acid transport and metabolism; [G] Carbohydrate transport and metabolism; [I] Lipid transport and metabolism; [J] Translation, ribosomal structure and biogenesis; [L] Replication, recombination and repair; [M] Cell wall/membrane/envelope biogenesis; [N] Cell motility; [O] Posttranslational modification, protein turnover, chaperones; [R] General function prediction only; [S] Function unknown; [U] Intracellular trafficking, secretion, and vesicular transport.

Given that the CcpA regulon includes numerous transcription factors, the CcpA transcriptome consists of genes that are directly regulated by CcpA and those that are secondarily affected by CcpA-inactivation, but there is limited understanding of the relative contribution of direct vs. indirect CcpA-mediated regulation which in turn obscures precise characterization of CcpA-DNA interaction. We sought to better define genes that are directly regulated by CcpA by determining the CcpA transcriptome in multiple GAS serotypes with the idea that deletion of a highly conserved regulator should impact a core set of genes across multiple serotypes. Thus, we expect the “core” to consist mostly of conserved *cre*-bearing primary targets of CcpA, and genes that are modulated by conserved CcpA-affected regulators. To this end, we performed RNAseq analyses in quadruplicate for the three serotype representatives and their Δ*ccpA* mutants grown to mid-exponential phase in THY. RNAseq data has been deposited in GEO repository under the accession #GSE84641. Consistent with the reproducibility of the data, principal component analysis proved the transcriptomes of the wild-type and their *ccpA*-inactivated strains to be distinct in each serotype (Supplementary Fig. S4). We defined differentially expressed genes as those with a minimum mean transcript level difference of 2-fold and a corrected *P* value of ≤0.01. When analyzing genes that have homologs in all three serotypes, we observed significantly different transcript levels for 310, 386 and 307 genes in the *ccpA* mutants compared to their parental M1, M3 and M28 serotype strains respectively (Fig. 3a). This amounted to an average of 19% of ORFs in each serotype being differentially expressed, which is comparable to the observed CcpA regulon in *Staphylococcus aureus* (16%) and *Streptococcus pneumoniae* (14–19%)^15^,^27^. 173 genes were differentially expressed in all three serotypes thereby defining the core GAS CcpA regulon (Fig. 3b; Supplementary Table S1). Importantly, the core genes account for only 32% of the genes differentially expressed among the three serotypes indicating that our multi-serotype approach helped to remove serotype-specific genes that are unlikely to be directly regulated by CcpA. Supporting our hypothesis that the GAS CcpA core regulon was enriched for primary CcpA target genes, nearly 90% of the core genes were CcpA-repressed. This contrasted with an average of 63% repressed : 37% activated ratio when a particular serotype was studied (*P* < 0.001 by χ^2^ test; Supplementary Table S2). Of the 250 genes differentially regulated in only a single serotype, which accounted for 46% of all genes affected by CcpA-inactivation, only 35% were repressed by CcpA while 65% were activated. These findings suggest that much of CcpA-mediated activation, which has accounted for up to 40% of CcpA-influenced genes in previous investigations^42^, is unlikely to be a direct effect of CcpA, but rather to strain-specific regulatory circuits influenced by CcpA-inactivation. These data are in concert with those published in *B. subtilis* which identified only three operons as activated by CcpA^43^.

To better understand the functional impact of the CcpA regulon across serotypes, we assigned genes into cluster of orthologous groups (COGs). Five COGs were consistently enriched in all three serotypes of which carbohydrate transport and metabolism [G] comprised the largest number of genes (Fig. 3c). Two of these COGS, G and S, have also been reported to dominate the CcpA-influenced genes in other gram-positive pathogens^20^,^27^,^31^,^42^. However, several COGs, such as M (Cell wall/membrane/envelope biogenesis), R (General function prediction only) and E (Amino acid transport and metabolism), that had previously been identified as part of the CcpA regulon in other species^31^,^42^ were only enriched in one or two of the GAS serotypes studied here suggesting that these particular COGs may be part of the strain-specific CcpA regulon rather than a core aspect of CcpA physiology.

Given the critical impact of CcpA on GAS pathogenesis, we determined how CcpA-inactivation affected known virulence gene transcript levels. Interestingly, only the nine gene operon encoding the cytolysin, streptolysin S (*sagA-sagI*)^44^, was influenced by CcpA in every serotype (Supplementary Fig. S5). CcpA-dependent serotype-specific variation was observed for the IL-8-degrading *Streptococcus pyogenes* cell envelope proteinase (SpyCEP) and the immunoglobulin cleaving endoglycosidaseS (EndoS) (Supplementary Fig. S5)^45^,^46^. The promoter regions of the *spyCEP* and *endoS* genes contained no clear distinctions to account for the serotype-specific differences. Taken together, we conclude that the multi-serotype transcriptomic approach allowed for clarification of the core CcpA regulon.

### Functional *cre* sites are highly conserved across GAS serotypes

**Figure 4:**
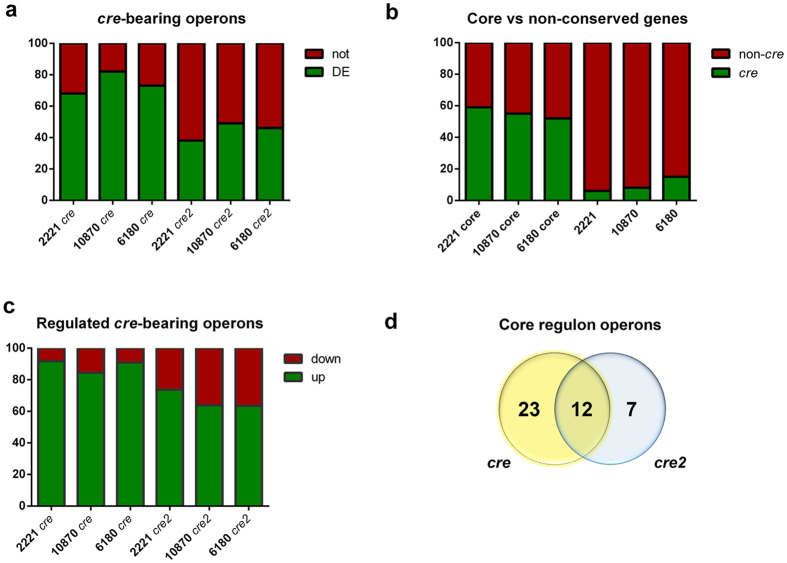
Distribution of *cre* and *cre2* motifs. (**a**) Percentage of *cre* and *cre2*-bearing operons that exhibit CcpA-dependent differential expression (DE) or do not (not), in the three GAS serotype strains. (**b**) Distribution of the core regulon genes based on the presence (*cre*) or absence (non-*cre*) of a *cre* site in the promoter. (**c**) Differentially regulated genes containing *cre* or *cre2* operators displayed by relative percentages that are repressed (up) or activated (down) in Δ*ccpA*. (**d**) Number of *cre* and *cre2* sites in the GAS core CcpA regulon depicted as a Venn diagram.

Although we postulate that serotype-specific CcpA-affected genes primarily represent indirect effects, it remains formally possible that the observed differences could be due to heterogeneity in *cre* site composition between homologs in the various serotypes (e.g. while the promoter of a gene specifically regulated in MGAS2221 contains a *cre* site, this site is absent in MGAS10870 and MGAS6180). We searched for *cre* sites within 150 bp upstream and 50 bp downstream of predicted translation start sites for all genes (only the first gene for operons) in MGAS2221, MGAS10870 and MGAS6180. We identified 72, 71 and 75 *cre* sites respectively, of which 68, 82 and 74% were differentially regulated by CcpA as demonstrated by RNA-seq (Fig. 4a; Supplementary Table S3). Of the 173 core genes, 103, 95 and 90 genes in serotypes M1, M3 and M28 respectively were present in operons with *cre* operators. In contrast, only 4, 10 and 11 of the 250 genes that displayed serotype-specific alteration in M1, M3 and M28 strains had *cre* sites (Fig. 4b). Consistent with our core transcriptome data indicating that CcpA mainly functions as a repressor, 92, 84 and 90% of the regulated *cre*-bearing genes were derepressed in the *ccpA* mutants of M1, M3 and M28 respectively (Fig. 4c). These data support the idea that serotype-specific variation as well as CcpA-mediated gene activation in GAS is primarily driven by indirect targets of CcpA.

### Comparison of *cre* and *cre2* sites in the GAS CcpA regulon

Although the vast majority of CcpA-DNA interaction has focused on *cre* sites^12^, a recent study assessing the targets of CcpA in *S. suis* reported a second CcpA-binding DNA motif, designated as *cre2* 42. Given that the CcpA protein in GAS and *S. suis* is highly conserved (79% identity), we next sought to determine the occurrence of *cre2* sites in our representative GAS strains. A consensus motif developed from 45 *cre2* sites reported in *S. suis* was used to search the regulatory region of the first genes of operons in MGAS2221, MGAS10870 and MGAS6180. Approximately 100 putative *cre2* sites were identified in each serotype (Supplementary Table S4); nearly twice that reported in *S. suis.* The proportion of *cre2*-containing genes that had differential transcript levels were significantly lower compared to *cre*-bearing genes (P < 0.001, Fig. 4a). Moreover, genes with *cre2* operators had a significantly lower frequency of CcpA-dependent repression than *cre*-containing genes (P < 0.001, Fig. 4c). The core GAS CcpA regulon has only 19 *cre2*-bearing operons compared to 35 *cre*-bearing operons, with 12 operons containing both (Fig. 4d). Thus, although *cre2* sites might be involved in mediating CcpA-based regulation in a different growth phase or metabolic status of the cell, our data suggest that *cre* sites predominate in determining the CcpA regulon under the tested conditions.

### Developing an improved *cre* motif

**Figure 5:**
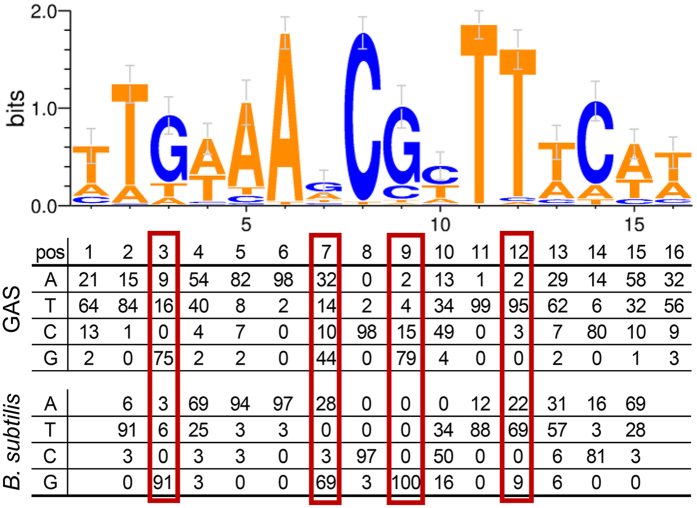
The improved GAS *cre* motif. Weblogo representation of the GAS *cre* consensus motif. The frequency of occurrence of nucleotides for all core GAS cre sites is compared to that reported for *B. subtilis*^43^. Positions displaying notable differences between *S. pyogenes* and *B. subtilis* are marked with red boxes.

To this point, we had more clearly defined the core GAS CcpA regulon through our multi-serotype transcriptomic approach which facilitated removal of off-target effects that pervaded previous single strain studies. Thus, we next sought to use these data to optimize a *cre* consensus for GAS in particular and pathogenic gram-positive bacteria in general. Importantly, the *cre* site consensus derived from our GAS CcpA core regulon had several key differences from the *B. subtilis* motif (Fig. 5). A recent study reported a near absolute occurrence of Cytosine (C) and Guanine (G) at positions 8 and 9 in *B. subtilis*^43^. In GAS, the occurrence of C8 is also nearly absolute, but position 9 shows more flexibility, with almost 20% of the regulated genes coding for a nucleotide other than G at this position. The predominance of G at position 7 for *B. subtilis* is not observed for GAS, which showed greater variation (Fig. 5). There is also increased flexibility at position 3 in terms of its requirement of a G in GAS. In contrast, the requisite of a Thymine (T) at positions 11 and 12 is much more stringent in GAS compared to *B. subtilis*. The amino acid residues in the CcpA protein identified as interacting with these specific *cre* base pairs in a crystallographic analysis are highly conserved within the CcpA subfamily^12^ and show no differences between *S. pyogenes* and *B. subtilis*. However, there is increased amino acid variation between CcpA from GAS and *B. subtilis* in the C-terminal region, which has been shown to interact with the N-terminal DNA binding domain, that may account for the observed heterogeneity in *cre* site composition.

**Figure 6:**
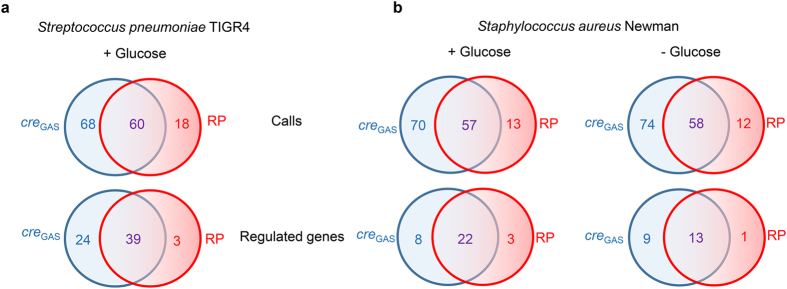
Prediction of *cre* operators in other gram-positive pathogens. The suite of *cre* sites predicted for (**a**) *S. pneumonia* and (**b**) *S. aureus* using the *cre*_GAS_ and by RegPrecise (RP) are compared and displayed as Venn diagrams (calls). The RegPrecise database was accessed in November, 2015. *Cre* sites that have reported experimental evidence of CcpA-dependent expression are displayed in the lower panel (regulated genes).

Next we sought to determine whether the GAS-optimized *cre* consensus (denoted as *cre*_GAS_ for the remainder of this manuscript) improves the ability to identify CcpA-regulated genes in major pathogenic gram-positive bacteria with published CcpA transcriptomes^15^,^21^. To this end, *cre*_GAS_ sites were predicted for *S. aureus* and *S. pneumoniae* and then compared to their published CcpA regulons to determine how many of our predicted *cre*_GAS_ sites have experimental evidence of CcpA-dependent gene expression changes. We then performed a similar comparison for the *cre* sites of *S. aureus* and *S. pneumonia* predicted by the RegPrecise^47^ database, which uses *in sillico* methods to curate regulons of various transcription factors. In *S. pneumoniae*, we predicted 60 sites in concert with RegPrecise, of which 39 (65%), have been confirmed to be regulated experimentally^15^ (Fig. 6a). We identified 68 sites not called by RegPrecise, of which 24 (35%) had reported CcpA-dependent regulation^15^. Among these were genes encoding an arginine deiminase and a neuraminate lyase, both of which contribute to *S. pneumoniae* virulence^48^,^49^. In contrast, of the 18 cre sites predicted by RegPrecise alone, only 3 (17%) were affected by CcpA-inactivation^15^. Similarly, in *S. aureus* we identified 17 *cre*_GAS_ sites that are present in CcpA-affected genes^27^ but were not predicted by RegPrecise whereas RegPrecise alone identified only 4 such sites (Fig. 6b). Hence, our *cre*_GAS_ consensus derived from our multi-strain approach proved to be a powerful tool to refine understanding of the CcpA regulons of pathogenic gram-positive organisms.

## Conclusions

Herein, we employed a multi-serotype approach to clarify the core GAS CcpA regulon, which we found to mainly consist of repressed genes that are likely to be directly affected by CcpA given the presence of *cre* elements. The power of this method is exemplified by the fact that only 173 of the 540 genes (32%) differentially regulated in the three serotypes were affected in all three strains. Thus, our data suggest that many of the previously published CcpA transcriptomes are likely to contain strain-specific, secondary effects of CcpA-inactivation. Our improved *cre*_GAS_ motif derived from the multi-strain transcriptome data should facilitate more accurate *in sillico* prediction of CcpA-regulated genes in a broad array of pathogens.

Interestingly, we observed that a significant proportion of the CcpA core regulon (55%) is non-*cre*-bearing. This observation could indicate an undiscovered, non-*cre*-based mode of CcpA regulation, such as the *cre2* motif recently proposed in *S. suis*^42^. However, even incorporation of the *cre2* motif accounted for only 15 additional genes in the core regulon. It remains possible that the core GAS CcpA regulon genes that lack *cre* or *cre2* sites are part of the transcriptional regulatory network of a CcpA-affected regulator, given that the core CcpA regulon includes at least two TCSs, eight stand-alone regulators, and two transcriptional antiterminator proteins (Supplementary Table S1).

The concept of metabolic regulators as virulence determinants is supported by the fact that the ability of a pathogen to thrive requires the capacity to exploit food sources in the host, which in turn relies on control of metabolic pathways. Thus, further understanding of CcpA function could assist in the development of novel preventive or therapeutic strategies applicable to numerous gram-positive pathogens.

## Methods

### Ethics Statement

Mouse experiments were conducted as per protocols approved by the MD Anderson Institutional Animal Care and Use Committee (Protocol Number: 00000808). All efforts were made to minimize suffering.

### Bacterial Strains, media and growth

All strains used in this study are listed in Table S5. Group A *Streptococcus* (GAS) strains were routinely grown in Todd-Hewitt media supplemented with 0.2% yeast extract (THY) at 37 °C with 5% CO_2_. Chloramphenicol (4 ug/ml) and spectinomycin (150 ug/ml) were used to select for the CcpA-complementing and CcpA-inactivating plasmids respectively. Non-polar insertional mutagenesis was employed to obtain isogenic *ccpA* mutants in each of the serotype strains as previously described for MGAS2221^39^. The *ccpA* gene from each serotype strain was used to create their respective CcpA-complementing plasmid as described previously^39^ and introduced into these mutants for genetic complementation. Mutants and complemented strains were verified by Southern blots and qRT-PCR of *ccpA* and known CcpA-regulated genes (data not shown) as described previously^50^.

### Animal Studies

Twenty five female outbred CD-1 Swiss mice (Harlan-Spraigue-Dawley) were used for each GAS strain for the bacteremia^51^ and the myositis^52^ models respectively. Mice were injected intraperitoneally (IP, bacteremia) and intramuscularly (IM, myositis) with 1 × 10^7^ CFUs of MGAS2221 and MGAS10870 strains and their derivatives. In keeping with the less virulent nature of strain MGAS6180, we used 2 × 10^8^ and 3.5 × 10^8^ CFU for the IP and IM models respectively. Mice were observed until no deaths had occurred for 72 hrs in any group. Kaplan-Meier survival analysis was used to compare the near-mortality rates and differences were considered statistically significant for a P < 0.05 after considering multiple comparisons. For the oropharyngeal model^53^, 35 mice were used for each of the GAS strains. We inoculated 1 × 10^7^ CFUs of MGAS2221 and MGAS10870 and their derivative strains, whereas 2 × 10^8^ CFUs were used for MGAS6180 and its derivative strains. The throats of all mice were swabbed before inoculation and daily after to determine GAS CFU density over time. For skin/soft tissue infections, immunocompetent hairless SKH1-hrbr female mice (Charles River BRF) were used, because the lack of hair facilitates lesion monitoring and excision. Twenty mice were used for each GAS strain tested and inoculated subcutaneously with 1 × 10^7^ CFU of MGAS2221 and MGAS10870 and their derivative strains and 4 × 10^8^ CFU MGAS6180 and its derivative strains. Development of ulcers were monitored and lesion sizes were measured daily until healing. Groups were compared using a Two Way ANOVA and considered significant for a P < 0.05. All statistical analyses were performed using GraphPad Prism software. To ensure that observed phenotypic differences were not due to spurious mutations, we included the complemented strains in the bacteremia model. Given that complementation led to a restored phenotype in the bacteremia model, the complemented strains were not included in the remainder of the animal challenge studies in order to minimize animal numbers.

### Statistics

For the bacteremia and the myositis models of infection, a total of 25 mice per strain was chosen because this number provides an 80% chance of detecting a 50% change in mortality rate using a log-rank test power calculation. The power calculation was based on previous studies done using strain MGAS2221 in this model. A total of 35 animals per strain was used for the pharyngeal challenge studies as this number provides an 80% chance of detecting a 35% reduction in pharyngeal colonization rates for the isogenic mutant strains compared to wild-type. The power calculation is based on pilot dose-challenge studies done with strain MGAS2221 and prior investigations with other GAS strains using this model. For skin/soft-tissue infection model, a total of 20 mice per strain was chosen because this number provides an 80% chance to detect a 30% decrease in median lesion size between the two strains. The power calculation was based on a pilot dose-escalation study using strain MGAS2221 and previous studies done using strain MGAS2221 in this model.

No inclusion/exclusion was applied to the animals and no randomization was used. All investigators were blinded for all the infection models using letter codes for each group. The code was broken only after completion of all analysis. Assumptions of normal distribution were tested in GraphPad Prism using the Shapiro-Wilk normality test when appropriate and variance was estimated using the F test.

### Analysis of transcript levels

For RNA seq analysis, RNA was isolated from four replicate cultures from each strain grown to mid-exponential phase in THY (OD ~ 0.6) using the RNeasy kit (Qiagen) and processed as previously described^50^. Each Illumina FASTQ library was first processed by FASTX (version 0.0.6; http://hannonlab.cshl.edu/fastx_toolkit/download.html) to remove low complexity and low quality reads. EDGE-pro v1.3 (Estimated Degree of Gene Expression in Prokaryotes) software^54^, was then used to align the reads using Bowtie2 v2.1.0 and estimate gene expression directly from the alignment output. For MGAS2221 and MGAS6180 strains, FASTA of the references genome sequences (.fna), protein translation table with coordinates of protein coding genes (.ptt), and a table containing coordinates of tRNA and rRNA genes for each strain was obtained from the NCBI database (ftp://ftp.ncbi.nih.gov/genomes/Bacteria/Streptococcus_pyogenes_MGAS6180_uid58335/; ftp://ftp.ncbi.nih.gov/genomes/Bacteria/Streptococcus_pyogenes_MGAS5005_uid58337/). As the MGAS2221 genome is not publicly available, we used the MGAS5005 genome, which is identical to MGAS2221^38^ in gene content. For the MGAS10870 strain, the.ptt and.rnt files were generated from an in house genome assembly and were used as inputs into EDGE-pro. Gene names in MGAS10870 correspond to the previously defined open reading frames from the fully sequenced strains MGAS315. EDGE-pro was run with default parameters to generate a RPKM value table, except for defining the read length as 50 bp and using 64 threads (-t 32) on a Dell PowerEdge R910 (32-Core, 1Tb ram). RSeQC program was used for QC metrics and alignment statistics^54^. Samples were concatenated through edgeToDeseq.perl script provided by EDGE-pro. DESeq^55^ was used to identify the gene expression level (http://www.r-project.org). Differentially expressed genes between the wild-type and Δ*ccpA* strains were considered significant based on a *p-*value ≤ 0.01. Over-represented COG group of differentially expressed genes were calculated by Fisher Exact test (*p-*values > 0.05) using in-house R script. Gene Ontology (GO) enrichment was performed by Gorilla^56^.

For Taqman real-time qRT-PCR, strains were grown in duplicate on two separate occasions to mid-exponential phase in THY and processed as described previously^50^. The gene transcript levels between the wild-type and *ccpA*-inactivated derivative of each serotype strain was compared using an ordinary one way ANOVA. Primers and probes used are listed in Table S5.

### *In silico* CcpA regulon identification

The CcpA regulon was predicted *in silico* using a classical probabilistic weight matrix strategy^57^. Briefly, we considered an operon as a cluster of genes encoding in the same direction with less than 50 base pairs between them^58^. We generated two independent CcpA binding site consensus weight matrices according to previously reported data^42^,^43^. Using the algorithm MAST^59^ each of the matrices was used to screen 150 bp upstream and 50 bp downstream of each operon contained in MGAS2221, MGAS6180 and MGAS 10870 strains of *S. pyogenes* genomes. Finally, we accepted only those CcpA binding site predictions which presented a similarity higher than 70% with the corresponding consensus matrix. Using the predicted CcpA binding site sequences conserved in the core regulon, we generated the new consensus matrix, *cre*_GAS_ and screened the complete genome of *S. pneumoniae* TIGR4 (NC_003028.3) and *S. aureus Newman* (NC_009641.1).

## Additional Information

**How to cite this article**: DebRoy, S. *et al.* A Multi-Serotype Approach Clarifies the Catabolite Control Protein A Regulon in the Major Human Pathogen Group A *Streptococcus. Sci. Rep.* **6**, 32442; doi: 10.1038/srep32442 (2016).

## Supplementary Material

Supplementary Information

Supplementary Table S1

Supplementary Table S3

Supplementary Table S4

Supplementary Table S5
