# Supplementary material for: A Multi-Serotype Approach Clarifies the Catabolite Control Protein A Regulon in the Major Human Pathogen Group A Streptococcus
Source: Sci Rep. 2016 Sep 1;6:32442. doi: 10.1038/srep32442 (PMC5007534; doi:10.1038/srep32442)
Supplement: Supplementary Information [file srep32442-s1.pdf]

Supplementary Information

A Multi-Serotype Approach Clarifies the Catabolite Control protein A Regulon in the Major Human Pathogen Group A *Streptococcus*

Sruti DebRoy[a], Miguel Saldaña[a], Dante Travisany[d,e], Andrew Montano[a], Jessica Galloway-Peña[a], Nicola Horstmann[a], Hui Yao[b], Mauricio González[e,f], Alejandro Maass[d,e,g], Mauricio Latorre[d,e,f] and Samuel A. Shelburne[a,c, *].

[a]Department of Infectious Diseases, MD Anderson Cancer Center, Houston, Texas, USA

[b]Department of Bioinformatics and Computational Biology, MD Anderson Cancer Center, Houston, Texas, USA

[c]Department of Genomic Medicine, MD Anderson Cancer Center, Houston, Texas, USA

[d]Mathomics, Center for Mathematical Modeling, Universidad de Chile, Beauchef 851, 7th Floor, Santiago, Chile.

[e]Center for Genome Regulation (Fondap 15090007), Universidad de Chile, Blanco Encalada 2085, Santiago, Chile.

[f]Laboratorio de Bioinformática y Expresión Génica, INTA, Universidad de Chile, El Líbano 5524, Macul, Santiago, Chile.

[g]Department of Mathematical Engineering, Universidad de Chile, Beauchef 851, 5th Floor, Santiago, Chile.

*corresponding author : sshelburne@mdanderson.org

Figures

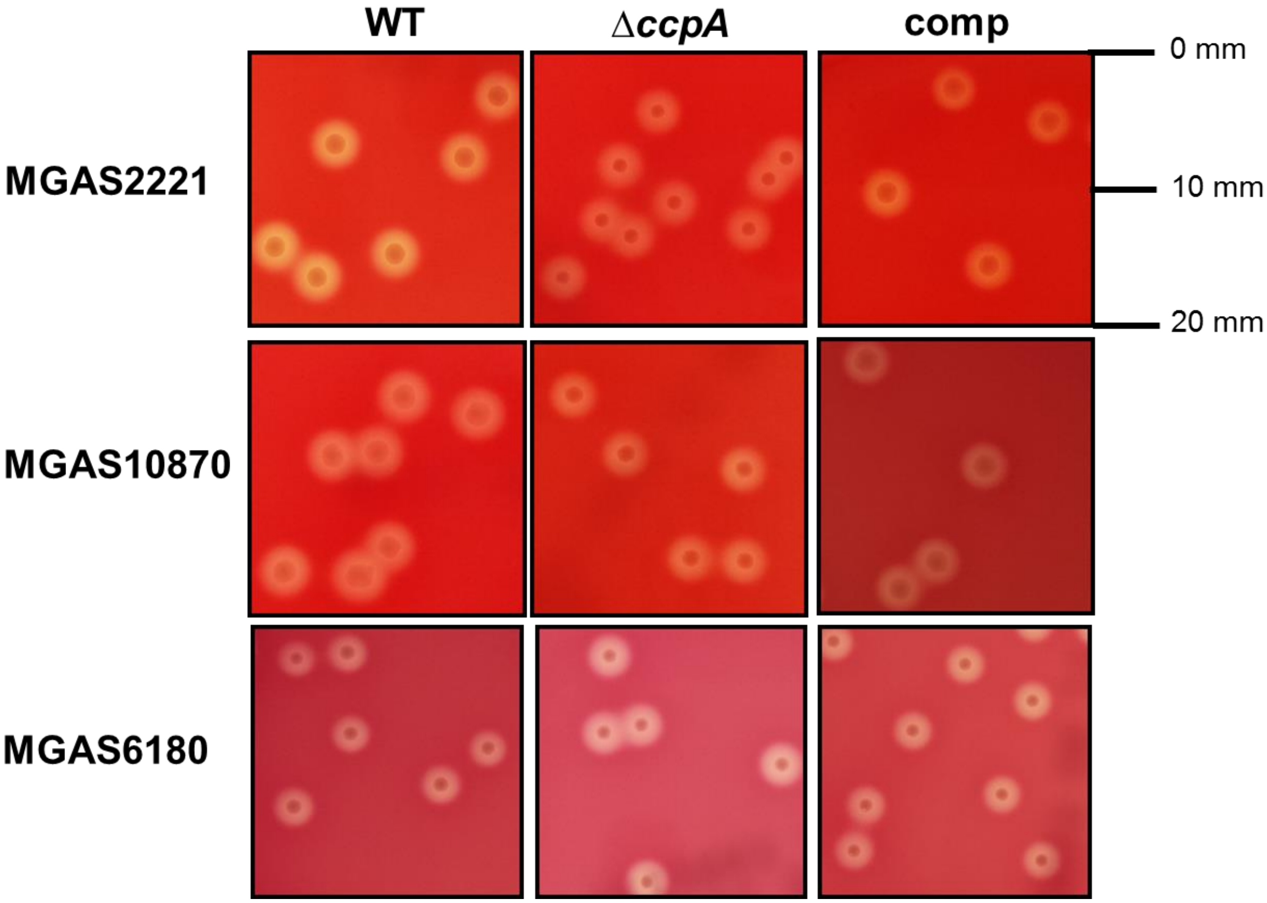

Supplementary Figure S1. Colony Morphology of GAS serotype strains. Wild-type (WT) serotype strains along with their *ccpA*-inactivated (Δ*ccpA)* and complemented (comp) strains were grown overnight in THY and plated on 5% sheep blood agar plates. Colonies were observed and recorded after overnight incubation. Experiment was repeated on at least two independent occasions.

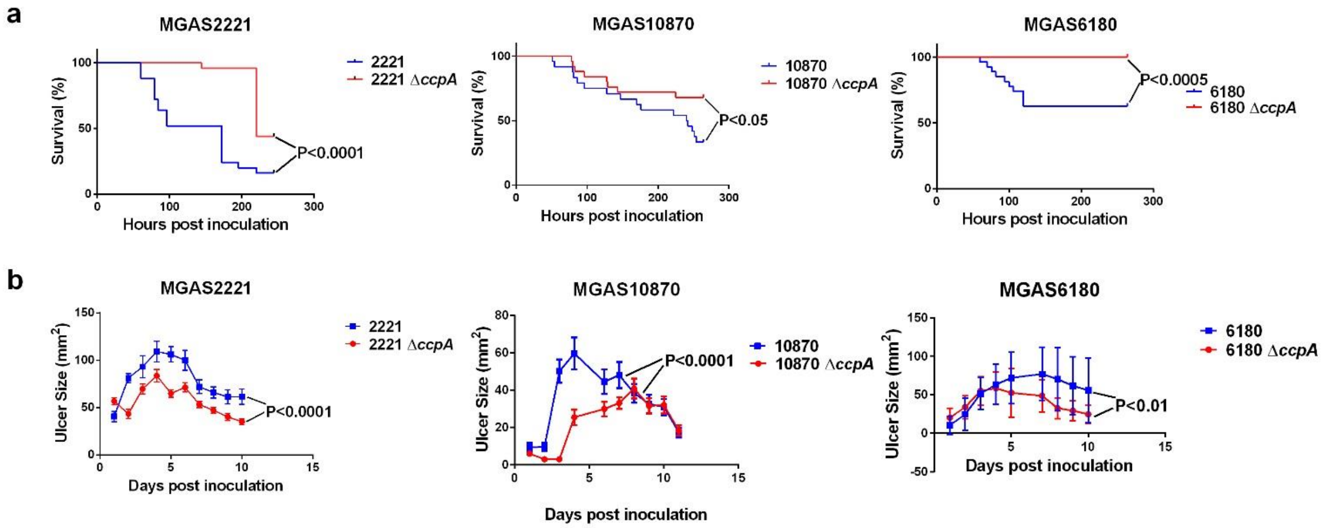

Supplementary Figure S2. Effect of CcpA deletion on virulence of GAS serotype strains. CD-1 swiss mice were inoculated with GAS strains by intramuscular route and monitored to near-mortality for the myositis model of infection (a) and survival was graphed. For the subcutaneous model (b), immunocompetent hairless SKH1-hrbr female mice were injected. Ulcer size was measured until healing and plotted.

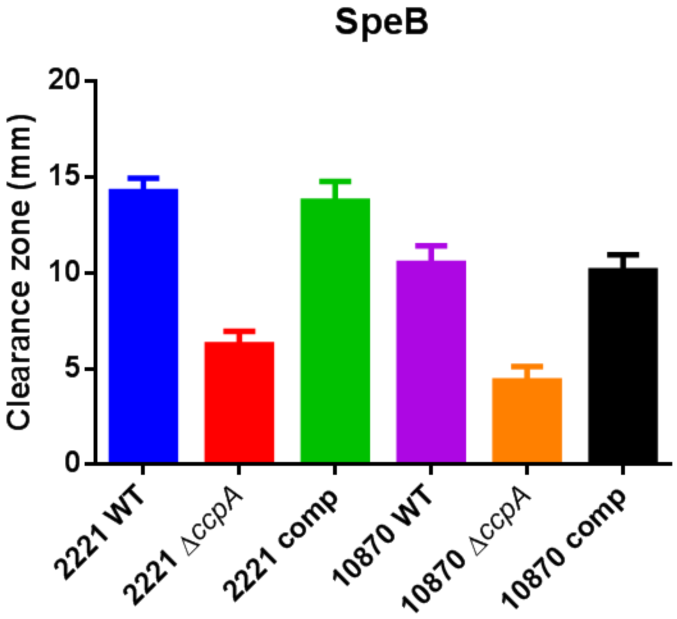

Supplementary Figure S3. Quantification of SpeB activity. Strains were grown overnight on 5% sheep blood agar plates and then inoculated into milk plates, as described previously[14]. Caesin hydrolysis, a marker of SpeB activity, was estimated by measuring the diameter of the clearance zones on milk plates. Experiment was performed using duplicate cultures on two independent occasions and the mean and standard deviations were plotted. No activity was observed for MGAS6180.

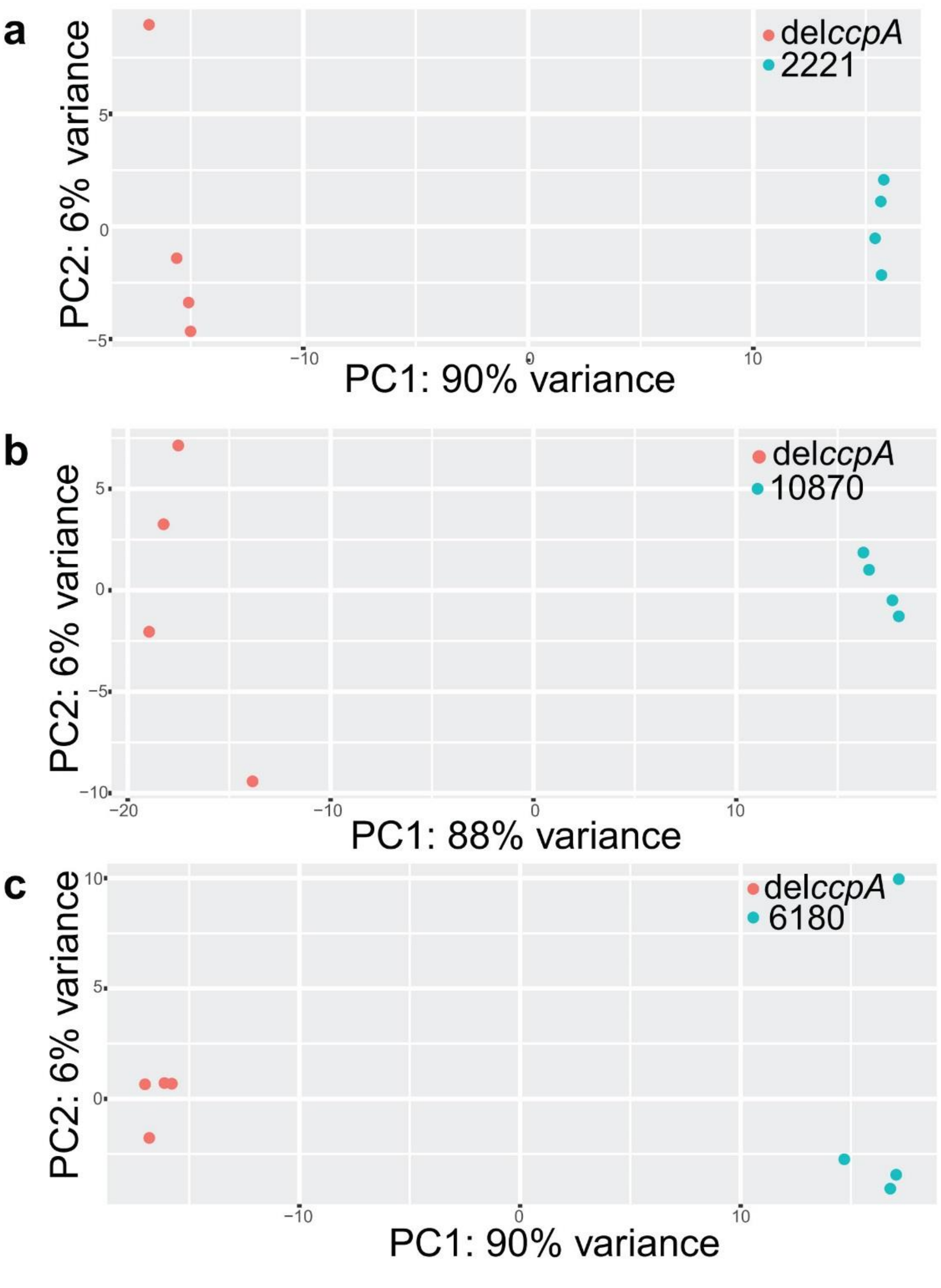

Supplementary Figure S4. Principal Component Analysis. Principal component analysis showing that the wild-type and *ccpA*-inactivated strains of (a) MGAS2221, (b) MGAS10870, and (c) MGAS6180 are clearly distinct based on the transcriptomes.

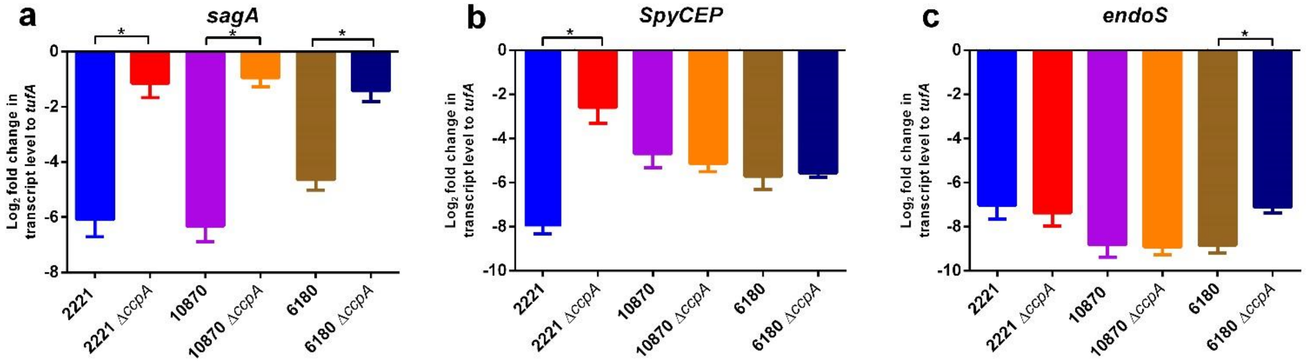

Supplementary Figure S5. Analysis of virulence gene transcript levels. Taqman quantitative real-time PCR was used to verify the alteration in the transcript levels of virulence genes in the core CcpA regulon. Data shown are the mean ± standard deviation of 8 data points. * indicates a statistically significant difference between the wild-type and *ccpA*-inactivated derivative of a serotype strain.

Tables

Supplementary Table S1: The core GAS regulon.

Supplementary Table S2. Proportions of up and down-regulated genes in individual GAS serotypes in comparison to that in the core regulon.

Supplementary Table S3: List of cre sites identified in MGAS2221, MGAS10870 and MGAS6180

Supplementary Table S4: List of cre2 sites identified in MGAS2221, MGAS10870 and MGAS6180

Supplementary Table S5. Strains, plasmids and primers used in this study.

Supplementary Table S2. Proportions of up and down-regulated genes in individual GAS serotypes in comparison to that in the core regulon.

	Genes upregulated in Δ*ccpA* (%)	Genes downregulated in Δ*ccpA* (%)
Core regulon	90	9
MGAS2221 only	41	59
MGAS10870 only	20	80
MGAS6180 only	43	57
